# Psychosocial issues of neonatal screening in the context of its major expansion: a scoping review

**DOI:** 10.3389/fpsyg.2025.1564032

**Published:** 2026-03-05

**Authors:** Anne-Laure Sébert, Marcela Gargiulo, Pascale De Lonlay, Jean-Baptiste Arnoux, Daniel Vaiman, Céline Bensimon, Marco Araneda

**Affiliations:** 1 Research Center for Psychoanalysis, Medicine and Society (EA 3522), Labex “Who am I?”, Department of Psychoanalysis, Institute of Humanities, Sciences and Societies, University Paris-Cité, Paris, France; 2 Laboratory of Clinical Psychology, Psychopathology, and Psychoanalysis (UR 4056), Institute of Psychology, University Paris-Cité, Paris, France; 3 Department of Neuromyology, Pitité-Salpêtrière Hospital Group, Assistance Publique-Hôpitaux de Paris, Institute of Myologie, Paris, France; 4 Reference Center for Inherited Metabolic Diseases, Necker Enfants Malades Hospital, Assistance Publique–Hôpitaux de Paris, INEM, Filière G2M, Paris, France; 5 MetabERN (European Reference Network for Hereditary Metabolic Disorders), Paris, France; 6 INSERM-1151, INEM, Paris, France; 7 Cochin Institute, U1016 INSERM, UMR 8104 CNRS, University Paris-Cité, Paris, France

**Keywords:** newborn screening, genetic disease, genomic medicine, psychosocial issues, scoping review, NBS

## Abstract

**Background:**

Newborn screening (NBS) programs began in the 1960s in the US and Europe. Systematically offered at birth, these programs enable the early detection of serious, rare, inherited diseases, facilitating timely treatment and improving survival rates. The range of detectable diseases has expanded significantly, with inclusion criteria evolving since the programs’ inception. Recent advances in genomics now allow for the detection of all DNA variant, enabling the identification of conditions typically diagnosed later in life and/or for which no effective treatment or preventive interventions are currently available. This expansion raises ethical and psychosocial concerns.

**Objectives:**

To explore the psychosocial challenges associated with the expansion of NBS.

**Methods:**

Between May 2022 and March 2024, a global scoping review was conducted using three databases and gray literature. PRISMA guidelines were followed, and thematic analysis was applied to synthesize findings.

**Results:**

Of 623 articles identified between 1997 and 2024, 68 met inclusion criteria, with nine additional gray literature references, for a total of 77 publications. Most studies originated from North America (*n* = 45) and Europe (*n* = 26), predominantly in healthcare sciences (*n* = 41), particularly medicine (*n* = 29), and in humanities and social sciences (*n* = 23), especially psychology (*n* = 14). The literature mainly addressed parents’ experiences during the early years following an abnormal NBS result; few studies explored healthcare professionals’ or patients’ perspectives. Three core thematic categories were identified: (1) parents’ experience of abnormal results and the impact on parent–child relationships; (2) strategies to mitigate psychosocial risks, including professional and public education; and (3) challenges related to NBS expansion.

**Discussion:**

These themes were interpreted as higher-level psychosocial constructs: (1) anxious and depressive dimensions of parental responses; (2) Vulnerable Child Syndrome as a construct shaping parental perceptions and caregiving practices; and (3) psychosocial implications of NBS expansion. Parental anxiety and depression were the most studied outcomes, particularly following abnormal or false-positive results. Current professional training and public education appear insufficient given rapid NBS evolution. Viewing NBS as a continuous process within family–healthcare relationships may help mitigate psychosocial risks.

**Conclusion:**

Further research in psychology and social sciences is critical to better understand and address psychosocial risks, particularly for late-onset conditions and those without current treatment or prevention options.

## 1 Introduction

Newborn screening (NBS) enables the detection of diseases before the onset of symptoms, as early treatment can significantly improve health outcomes, and, in some cases, even the chances of survival (Tluczek et al., 2011; Health Resources and Services Administration, 2024). Performed in the days following birth, screening is systematically offered to parents of newborns. NBS is mandatory in some countries (USA, Italy) (Campbell and Ross, 2003), although parents may in certain cases opt out, and optional in others, such as France (Health Resources and Services Administration, 2024), where it is free, nationally organized, and shows an exceptionally high uptake rate (99.93% according to the 2023 CNDN report). In contrast, in some countries where NBS is optional and not publicly funded, coverage remains low (5–20%). NBS programs can include both biological tests (such as analyses on dried blood spots) and physiological tests (such as hearing screening or screening for congenital heart disease). The dried blood spots analysis, or Guthrie test, is a sensitive, specific and inexpensive screening method which aims to limit the number of false-positive and false negative results while maximizing the identification of “true positives” (TPs). The test involves collecting a few drops of blood from the newborn baby, placing them on a blotting paper, and sending them to specialized laboratories for analysis (Grob, 2019).

The first newborn screening program involving healthcare professionals was launched in the early 1960s in New York, before being extended to most American states in 1966 (Department of Health, Wadsworth Center, 2025). Europe’s first national screening programs emerged within the same year, starting in Ireland (Tluczek et al., 2022; Labrador Cañadas et al., 2021).

Since 1968, the World Health Organization (2024) has been guided by the principles of Wilson and Jungner, who put forth conditions for the inclusion of diseases in screening programs: (i) the diseases to be screened must constitute a major public health concern; (ii) they must have a form of treatment or prevention available to the general population which, if started before the appearance of first symptoms, can positively impact the natural progression of the disease (Pàmpols Ros et al., 2022; Campbell and Ross, 2003).

In the early 2000s, the use of a new technology, mass spectrometry, proved a considerable advancement. It enabled dozens of diseases to be identified with a single drop of blood at a lower cost than prior techniques (Levy, 2021; Lantos, 2019). It also improved the sensitivity and specificity of screening tests, allowing for NBS to be extended to diseases less common in the general population (<1/50,000 births). As a result, in 2008, the WHO broadened inclusion criteria for NBS programs (Pàmpols Ros et al., 2022) to include treatable but incurable diseases; those for which treatments are available which reduce or delay the onset of symptoms without eliminating them (Tluczek et al., 2022; Bailey et al., 2009; Campbell and Ross, 2003). This has led to an increase in the number of diseases included in screening programs, although this varies considerably from country to country. In the United States, the number has risen from 7 in 2002, to 18 in 2005, 43 in 2009 (Grob, 2019; Pàmpols Ros et al., 2022) and 60 in some states in 2024 (NBS Legislation, 2024).

Today, genomic medicine allows for rapid genome sequencing at a low cost, raising the question of how best to integrate this technique into NBS (Chien and Hwu, 2023; Tluczek et al., 2022; Grob, 2019; Ersig et al., 2023; Goldenberg et al., 2022; Stark and Scott, 2023). The possibility of analyzing hundreds, even thousands, of genes easily and all at once represents a major advance in genomic analysis. The question has shifted from determining to screen for to considering which diseases might be excluded from screening. This perspective could lead to a redefining of eligibility criteria for diseases included in the screening program. The integration of genome sequencing in NBS raises the question of screening for diseases which do not currently meet inclusion criteria.

Three types of diseases could then be considered: (1) those that are treatable and/or curable, for which effective treatments exist, making it possible to prevent the onset of disease symptoms or serious related consequences (such as phenylketonuria or hypothyroidism in the current program) (Carpenter et al., 2018; O'Connor et al., 2018); (2) those that are treatable but incurable, for which treatments significantly improve patients’ quality of life by alleviating symptoms or slowing disease progression, though they do not cure or alleviate symptoms entirely (currently including cystic fibrosis or sickle cell anemia) (Vailly, 2011; Tluczek et al., 2011); (3) and finally, untreatable and incurable diseases which lack effective available treatments, or late-onset pathologies, where early detection offers no immediate health benefits for the child (Ersig et al., 2023; Van Dijk et al., 2021). This classification reflects a variable degree of actionability, i.e., the ability to take action by monitoring disease progression or implementing preventive measures, rather than a standpoint of pure curability (Pereira et al., 2021).

Some medical teams and patient associations are in favor of early, even presymptomatic diagnosis of untreatable and incurable diseases, to alleviate diagnostic delay and allow families to benefit from genetic counseling (GC) before a potential new pregnancy (Parsons et al., 2002).

Today there is a need to carefully consider broadening eligibility criteria for diseases included in screening while ethical and psychosocial issues are emerging in the literature, in order to assess the potential psychosocial, non-medical risks and benefits of this expanded screening (Hayes et al., 2007).

Against this backdrop, a great deal of research has emerged identifying the psychosocial risks that NBS may generate for children and their families, as well as practices to help limit them. Further research is needed to determine how to best support this evolving practice and anticipate any associated complications.

### 1.1 Purpose and objectives of a scoping review

In the context of the expansion of NBS, several studies have investigated psychosocial risks. Our primary aim is to conduct a scoping review of the significant results of this research in order to take stock of the current state of the literature, clarify key concepts, and analyze currently existing knowledge gaps (Munn et al., 2018).

Our secondary aim is to synthesize the literature to provide a critical perspective on the psychosocial implications of NBS for the child and his or her family, highlighting areas of uncertainty and offering insights that go beyond simply reporting aggregated findings. This review therefore seeks not only to map existing knowledge, but also to critically examine it in light of the expansion of NBS.

## 2 Methods

This scoping review was conducted by a multidisciplinary team of content and methods experts: ALS (postdoctoral researcher in psychology, member of the neonatal screening working group for neuromuscular disorders), MA (lecturer in psychology, also member of the neonatal screening working group), MG (professor of psychology, expert in genetics and myology, initiator of the neonatal screening working group), CB (PhD student in psychology with expertise in the neonatal screening program for phenylketonuria), DV (specialist in genomics and epigenetics), JBA and PDL (respectively medical doctors and heads of department in a pediatric unit involved in neonatal screening within a metabolic disease department). The review followed the PRISMA guidelines (Peters et al., 2015; Peters et al., 2017), with particular attention to the section dedicated to Scoping Reviews (PRISMA-ScR) (Page et al., 2021). The methodological framework was based on the approach developed by Arksey and O’Malley (2005), which comprises five key stages: (A) identifying the research question, (B) identifying relevant studies, (C) selecting studies, (D) charting the data, and (E) collating, summarizing, and reporting the results. The research team was actively involved in each stage of the process, from formulating the research question to data synthesis and interpretation, ensuring methodological rigor and a multidisciplinary perspective. In this review, we extended the final stage of Braun and Clarke’s thematic analysis by integrating a critical and interpretive perspective. Analytical comments are therefore interwoven throughout the Results section to highlight key questions raised by the synthesis. Because one of the main aims of this review was to examine how the notion of *psychosocial* has been conceptualized and evolved within the literature on the expansion of neonatal screening programs, no prior definition of “psychosocial” was imposed. To define inclusion and exclusion criteria, we applied the Population, Concept, Context (PCC) framework (Peters et al., 2015). Articles were eligible if they addressed psychosocial issues related to neonatal screening (NBS), regardless of the specific disease studied. Eligible diseases included those screened for in existing national programs or pilot studies worldwide, covering conditions linked to genes considered actionable to varying degrees, whether or not they can be detected through the Guthrie test. All types of research designs (qualitative, quantitative, and mixed methods) were included, provided they examined psychosocial aspects of NBS from the perspectives of the general population, screened families, healthcare professionals, and/or key stakeholders in public screening policies.

The keywords used in database searches are listed in Table 1. No place, date, type or language restrictions were applied.

**Table 1:** Keywords Boolean search strategies by database.

Databases	Keywords	Results
PubMed	(newborn screening) AND (psychosocial)	460
PsychInfo	(newborn screening) AND (psychosocial)	143
Cairn	(dépistage néonatal) et (psychosocial)	20
Total		623
Filters applied	No language, date, or publication type restrictions were applied. Articles retrieved were in English, Spanish and French, published between 1997 and 2024, and included various study types (research articles, essay, survey, reviews, reports, scientific syntheses, recommendations/legislations).	

Consistent with scoping review methodology, we also included gray literature (websites, institutional reports). Articles focusing solely on biological or medical aspects, without reference to psychosocial dimensions, as well as those dealing exclusively with physiological screening (pulse oximetry) or diseases not included in current or pilot NBS programs, were excluded.

### 2.1 Screening and selection process

**Figure 1:**
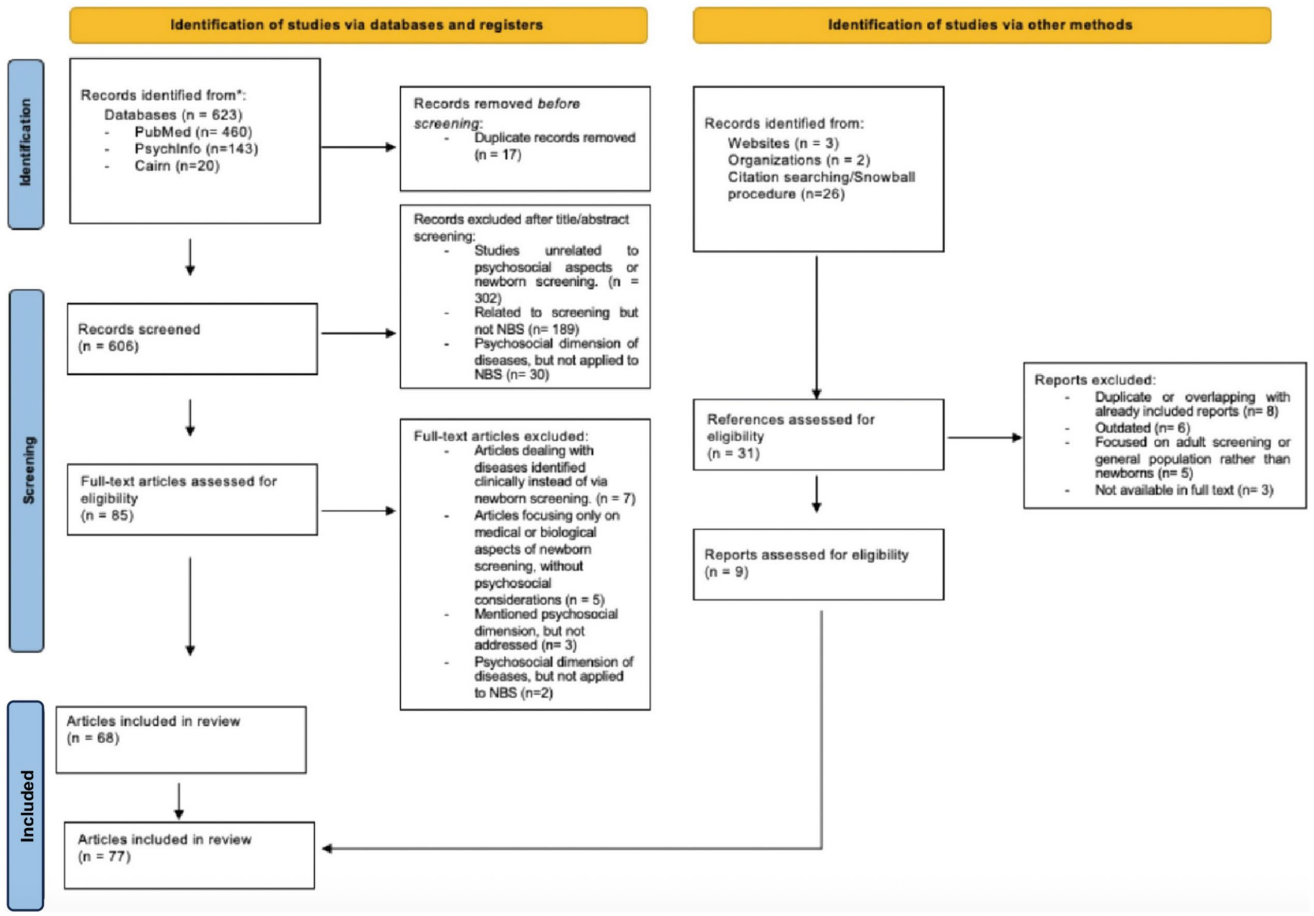
PRISMA search process of study inclusion and exclusion based on database searches and gray literature. The flow diagram was adapted from the PRISMA 2020 statement, Source: Page et al. (2021).

The review process was organized into multiple phases to ensure methodological rigor and transparency. A structured descriptive grid was used to extract data from each study. Methodological quality was assessed using the CROSS checklist for quantitative studies and the COREQ checklist for qualitative studies, with each criterion scored on a three-point scale (0–3). Two reviewers (ALS, MA) independently appraised each article, with discrepancies resolved through consensus meetings and, when necessary, arbitration by a third reviewer (MG). Regular multidisciplinary harmonization meetings ensured consistency and transparency across evaluations. Experts in genetics and/or clinical medicine (DV, PDL, JBA, and CB) subsequently reviewed the text, provided comments, and suggested reformulations to improve clarity and accuracy. Backward and forward citation tracking, key components of the snowballing method (Wohlin, 2014), were used to complemented the database searches, allowing the identification of additional relevant references and ensuring comprehensive coverage of the literature. Figure 1 illustrates the screening and selection process. This multi-phase, multidisciplinary approach enabled an iterative internal review process that strengthened both the psychosocial analysis and the overall scientific rigor of the review.

### 2.2 Data extraction

Data extracted from each article were stored and synthesized in separate Word documents. To ensure data accuracy, two team members (ALS and MA) independently extracted the information. The team met regularly to compare outputs, resolve discrepancies, and verify completeness.

The extracted variables included publication year, author details and disciplinary background, country, study population and sample size, disease screened, study design, objectives, main findings, and reported psychosocial dimensions.

### 2.3 Analysis and synthesis

In parallel with data extraction, we conducted thematic analyses following the six stages defined by Braun and Clarke (2006): (1) familiarization with data, (2) coding, (3) generating initial themes, (4) reviewing themes, (5) defining and naming themes, and (6) producing the final synthesis. This inductive approach allowed us to identify overarching categories and patterns emerging from the data (Smith et al., 2023; Kiger and Varpio, 2020; Thomas and Harden, 2008), while subthemes were incorporated to describe more specific psychosocial issues within each domain. Thematic analysis was chosen for its flexibility and suitability for synthesizing qualitative findings in scoping reviews (Braun and Clarke, 2006; Thomas and Harden, 2008). Both the study authors’ reported themes and participants’ quotations were analyzed, and we developed our own thematic structure. To guide the synthesis, we distinguished between primary themes (well-developed, frequently reported) and secondary themes (less-developed, less frequent), reflecting the relative emphasis in the dataset without imposing an external hierarchy. Regular harmonization meetings between ALS and MA (May 2022–March 2024) ensured coding consistency, interpretive coherence, and consensus on thematic definitions. Finally, gaps in empirical evidence were identified, and recommendations for future research on the psychosocial dimensions of neonatal screening were proposed. The Results are presented below, organized according to the main thematic domains emerging from this inductive analysis, each reflecting a distinct aspect of psychosocial issues in neonatal screening and its recent expansion.

## 3 Results

### 3.1 Search and screening results

The initial search conducted across PubMed, PsycINFO and CAIRN databases identified 623 articles published between 1997 and 2024. After removing duplicates, 606 unique records remained for screening. Following the multi-stage screening process, 68 articles met the inclusion criteria described above (Figure 1).

An additional nine references were identified through the *snowball method* (Wohlin, 2014), which involved screening the reference lists and citations of selected studies. These supplementary sources were added to the final corpus, bringing the total number of included articles to 77 (Supplementary Table 1).

All sections of this review (Introduction, Results, and Discussion) are based exclusively on the 77 articles included in the final corpus: 68 retrieved from database searches using predefined keywords (Table 1) and nine identified through gray literature sources. A number was assigned to each article included in the literature review, as shown in Supplementary Table 1, and these numbers are used consistently in the other tables. References are numbered from 1 to 23 and from 30 to 85. References cited in the Methods section are provided solely to describe methodological frameworks and analytical tools; they do not form part of the analyzed dataset.

The included articles were published across 12 countries, with a clear predominance of research originating from North America (58.4%, *n* = 45) and Europe (33.8%, *n* = 26). A smaller proportion came from Oceania (5.2%, *n* = 4) and Asia (2.6%, *n* = 2) (Table 2).

**Table 2:** Geographic distribution of identified articles.

Regions	Number of articles	Articles
North America	45 (58.4%)	
USA	40	14, 5, 33, 52, 82, 15, 76, 21, 9, 78, 37, 63, 10, 4, 61, 77, 70, 80, 81, 54, 38, 62, 1, 67, 65, 11, 71, 46, 40, 75, 68, 45, 73, 3, 49; 30, 12; 79; 69; 64
Canada	5	18, 48, 50, 44, 72
Europe	26 (33.8%)	
UK	11	57, 56, 17, 41, 31, 34, 36, 60, 74, 22, 84
France	7	47, 19, 42, 43; 58; 51; 85
Spain	2	8, 6
Netherlands	2	20, 32
Ireland	1	39
Italy	2	55; 59
Germany	1	83
Oceania	4 (5.2%)	
Australia	4	16, 35, 53, 23
Asia	2 (2.6%)	
Taiwan	1	13
China	1	66

Databases; Gray Literature.

Most studies focused on specific disorders screened through neonatal programs. The conditions most frequently represented were cystic fibrosis (36.4%, *n* = 28), sickle cell disease (11,7% *n*= 9), phenylketonuria (PKU) (5%, *n* = 4), Duchenne and Becker muscular dystrophies (DMD/BMD) (5%, *n* = 4), hearing loss (4%, *n* = 3), Pompe disease (4%, *n* = 3), spinal muscular atrophy (SMA) (4%, *n* = 3), lysosomal storage diseases (LSDs) (4%, *n* = 3), medium-chain acyl-CoA dehydrogenase deficiency (MCADD) (1.3%, *n* = 1), severe combined immunodeficiency (SCID) (1.3%, *n* = 1), congenital cytomegalovirus (CMV) (1.3%, *n* = 1), mucopolysaccharidoses (MPS) (1.3%, *n* = 1), and fragile X syndrome (FXS) (1.3%, *n* = 1). Additionally, 23 articles addressed newborn screening (NBS) in general, without focusing on a specific condition (Table 3).

**Table 3:** Conditions screened.

Conditions screened	Articles	Number, %
General NBS and gNBS	16 13; 14; 5; 8; 82; 15; 21; 6; 9; 10; 4; 70; 72; 66; 67; 46; 60; 61; 30; 12; 58; 79	23 (30%)
Cystic Fibrosis	35; 37; 63; 57; 56; 39; 18; 48; 44; 41; 54; 31; 38; 62; 1; 19; 55; 34; 71; 36; 40; 75; 68; 45; 73; 74; 84; 49	28 (36.4%)
Sickle Cell disease	62; 71; 68; 45; 41; 31; 38; 37; 63	9 (11.7%)
PKU	47; 17; 3; 69	4, (5%)
Duchenne Muscular Dystrophy/Becker Muscular Dystrophy	80; 11; 3; 22	4, (5%)
Hearing Loss	42; 65; 43	3, (4%)
Pompe Disease	33; 76; 77	3, (4%)
SMA	83; 53; 81	3, (4%)
Lysosomal Storage Disorders	52; 78; 59	3, (4%)
Untreatable disease	20; 51	2, (2.6%)
MCADD	50	1, (1.3%)
Severe Combined Immuno Deficiency (SCID)	32	1, (1.3%)
CMV (congenital cytomegalovirus)	65	1, (1.3%)
Mucopolysaccharidoses (MPS)	23	1, (1.3%)
Fragile X	11	1, (1.3%)
N/A	85; 64	2, (2.6%)

Databases; Gray Literature.

The healthcare sciences represented the majority of publications (*n* = 41), including medicine (*n* = 29), nursing (*n* = 9), biological sciences (*n* = 2), and genetics (*n* = 1). The humanities and social sciences accounted for 23 articles, primarily within psychology (*n* = 14). Population-based disciplines, including epidemiology, public health, and bioethics, were less represented (*n* = 13) (Table 4).

**Table 4:** Disciplinary field of articles.

Research field	Number of articles	Articles
Healthcare	41	
Medicine:	29	
General Medicine/Pediatric	12	37, 63, 57, 56, 54, 38, 62, 66, 71, 23, 45, 73
Genetic Medicine	11	16, 13, 33, 76, 9, 77, 48, 44, 46, 82; 51
Neurology	4	53, 80, 81, 83
Psychiatry	2	60, 69
Biological Science (biomedical)	2	68; 59
Genetic Science	1	78
Nursing	9	14, 5, 41, 1, 40, 74, 84, 22, 49
Humanities and Social-Sciences	23	
Psychology	14	35, 47, 17, 39, 18, 31, 42, 55, 11, 34, 36, 43, 75; 64
Sociology	4	4, 3, 61; 79
Ethics	3	8, 21, 20
Socio-anthropology	1	19
Philosophy	1	85
Population-Based	13	
Public Health	8	6, 32, 70, 67, 65; 30; 12; 58
Bioethics	3	52, 15, 10
Epidemiology	2	50, 72

Databases; Gray Literature.

Of the 77 articles included, 48 were empirical research studies. Among these, quantitative designs were the most frequent (*n* = 17), followed by qualitative studies (*n* = 14), mixed-methods studies (*n* = 10), and clinical studies (*n* = 7). The remaining publications consisted of literature reviews (*n* = 7), scientific syntheses (*n* = 7), institutional or policy reports (*n* = 5), essays (*n* = 4), recommendations (*n* = 4), and surveys (*n* = 2) (Table 5).

**Table 5:** Type of articles.

Type of articles	Number of articles	Articles
Research:	48	
Quantitative	17	83, 52, 21, 37, 63, 18, 80, 54, 38, 66, 62, 55, 23, 68, 45, 49, 71
Qualitative	14	33, 76, 20, 78, 17, 77, 50, 41, 81, 1, 19, 36, 3, 79
Mixed	10	32, 39, 48, 44, 43, 40, 53, 84, 22, 82
Clinical	7	57, 42, 75, 14, 51, 64, 69
Essay	4	47, 4, 61, 85
Survey	2	72, 67
Literature Reviews	7	35, 5, 10, 34, 46, 60, 74
Reports	5	15, 6, 56, 65, 73
Scientific syntheses	7	8, 16, 13, 9, 31, 11, 59
Recommendations/legislations	4	70, 30, 58, 12

Databases; Gray Literature.

Beyond this descriptive overview, the following section presents the thematic synthesis of the psychosocial issues identified across the selected studies. FP cases were the most frequently discussed paradigm in the literature, which is why they are used throughout the synthesis to exemplify recurring psychosocial processes.

### 3.2 Core categories identified from the data analysis

The results presented below are derived from the analysis of all 77 articles identified from the consulted databases and gray literature, as listed in Supplementary Table 1. The main themes emerging from this analysis are summarized in Table 6, which structures the following Results section. As previously noted in the Methods section, in this review we further expanded on the final stage by integrating our own critical and interpretive analysis.

**Table 6:** Core categories identified from the data analysis (The numbers in parenthesis refer to the reference list and indicate the studies contributing to each category or subtheme).

**Axe 1: Parents’ experience of receiving an abnormal screening result and its impact on their relationship with the child (30)** Unexpected news causes parental distress (5, 6, 8, 11, 17, 22, 31, 32, 33; 34, 35, 36, 37, 38, 39, 40, 41, 42, 43, 44, 45, 46, 47, 48, 49) The outcome of parental distress depending on the type of result: true positive, false positive, intermediate ◦ True positive NBS results (TP) (5, 17, 34, 35, 41, 50, 51, 52, 53, 54) ◦ Indeterminate, ambiguous, and inconclusive NBS results (4, 5, 48, 55, 56, 57) ◦ False-positive (FP) NBS results. (1, 2, 3, 4, 5, 6, 9, 10, 17, 19, 31, 34, 37, 38, 39, 45, 46, 50, 55, 58, 59, 60, 61, 62, 63, 64, 65, 66, 67, 68, 69, 70) Risk factors for negative psychosocial impacts, including Vulnerable Child Syndrome (VCS): parents’ difficulty in understanding NBS results delivered by healthcare professionals ◦ Ineffective transmission of information by doctors (FP cases used here as illustrative examples) (5, 17, 38, 45, 46, 62, 68) ◦ A lack of knowledge about NBS in the general population (5, 31, 32, 34, 40, 41, 44, 49, 52, 60) ◦ Negative perception of the healthcare professional-parent interaction (5, 34, 37, 38, 41, 42)
**Axis 2: Avenues and actions to limit the psychosocial risks associated with NBS: Education for professionals and the general population (8, 11, 32, 38, 39, 45, 46, 66, 68, 71, 72)** Education for professionals and the general public (8, 11, 32, 38, 39, 45, 46, 66, 68, 46, 71, 72) ◦ Professional training (4, 38, 45, 55, 68, 71) ◦ Information for the general public (5, 8, 11, 18, 34, 39, 41, 49 60, 73, 74) ◦ The limits of training in reducing the psychosocial risks associated with NBS in the context of its unprecedented expansion (6, 5, 8, 13, 32, 47) Information provided within the context of a dynamic, interactive relationship (5, 6, 8, 13, 32, 34, 36, 41, 42, 60, 75)
**Axis 3: The challenges of extending NBS (4, 5, 13)** « Patients in waiting »: a new category of patients driving changes in the care relationship (3, 4, 5, 6, 8, 9, 11, 4, 9, 22, 23, 33, 48, 52, 56, 70, 75, 76, 77, 78, 79, 80, 81, 82) Consequences of this new category of “patients in waiting” for healthcare systems (4, 8, 9, 10, 11, 13, 19, 43, 46, 70, 78, 79, 83) A new paradigm in healthcare (6, 9, 10)

The inductive thematic analysis identified three overarching axes, each encompassing several interrelated themes that capture the main psychosocial dimensions of neonatal screening as addressed in the literature (Table 6).

### 3.3 Axe 1: parents’ experience of receiving an abnormal screening result and its impact on their relationship with the child

In this review, we focus only on NBS results which fall outside of the expected norm. These results indicate that the child may be affected by one of the diseases being screened, and therefore are communicated to the parents. However, it is important to emphasize that NBS represents only the first step in the diagnostic process: screening is designed to overidentify infants who may be at risk. Consequently, most families who initially receive an abnormal result ultimately learn through subsequent diagnostic and clinical workup that their newborn does not have the disease. Abnormal results therefore require further testing to determine whether they are true-positive, false-positive or indeterminate (Health Resources and Services Administration, 2024). This disclosure can have significant psycho-social implications for families.

### 3.4 Unexpected news causes parental distress

When the screening result is abnormal, a healthcare professional (hospital doctor, hospital nurse, general practitioner, pediatrician, private nurse), who may or may not be known to the family, and who may or may not be specialized in genetic diseases, informs the parents of the need for further tests to confirm or rule out the diagnosis, either by phone or during a home visit (Bailey et al., 2009; Noke et al., 2014; Blom et al., 2021; Prakash et al., 2022). Quantitative research using numerical data and statistical analyses, shows that the disclosure of abnormal results and the wait for confirmation cause major parental distress (Tluczek et al., 2022; Duff and Brownlee, 2008; Blom et al., 2021; Li et al., 2023; Moran et al., 2007), often marked by symptoms of depression (Farrell et al., 2020a,b; Farrell and Christopher, 2008; Duff and Brownlee, 2008; Li et al., 2023), anxiety (Blom et al., 2021), panic, and insomnia (Quigley et al., 2018; Tluczek et al., 2005; Li et al., 2023).

Mixed and qualitative research attributes the intensity of this distress to the specific circumstances of the disclosure. In these cases, the disclosure concerns a baby who appears healthy, and shows no clinical signs of illness (Bailey et al., 2009; Chudleigh et al., 2016; Soriano, 2011; Le Driant et al., 2006; Li et al., 2023; Prakash et al., 2022). The disclosure is therefore particularly unexpected (Hayeems et al., 2016; Li et al., 2023), since the reassuring circumstances (a healthy-appearing baby) gave parents no reason to anticipate such news or to prepare themselves for it (Soriano, 2011). Moreover, the majority of parents have no recollection of the screening test, as it is often confused with other routine tests (Farrell et al., 2005; Campbell and Ross, 2003; Tluczek et al., 2005; Prakash et al., 2022), nor of having accepted to participate in NBS (Tluczek et al., 2022; Pàmpols Ros et al., 2022; Labrador Cañadas et al., 2021), even when doing so required written consent on their part (Chudleigh et al., 2016). After the first screening test, disclosure of results happens in the very first weeks after the birth of the child, and therefore in a context of high parental vulnerability (Carpenter et al., 2018; Soriano, 2011; Le Driant et al., 2006; Li et al., 2023; Hewlett and Waisbren, 2006).

Psychological studies point to the existence of a period of mourning for the imagined child: the healthy baby they thought they would have, full of dreams and promises, gives way to a sick infant (or one likely to be sick) who differs from the imagined ideal the parents had constructed. (Le Driant et al., 2006; Chudleigh et al., 2016; Soriano, 2011; Li et al., 2023; Prakash et al., 2022). This leads to considerable sadness, often associated with a strong sense of guilt (Chudleigh et al., 2016; Carpenter et al., 2018), or even shame (Bensimon and Araneda, 2022) for parents. Parental sadness may get in the way of adjusting to the infant’s specific needs (Soriano, 2011; Carpenter et al., 2018) and lead to relational disharmony at a key moment in the development of early parent–child bonds and the subject’s identity (Le Driant et al., 2006; Hayeems et al., 2017). This parental distress could therefore have long-term negative consequences on the child’s development (Tluczek et al., 2005; Bensimon and Araneda, 2022; Li et al., 2023; Carpenter et al., 2018). It appears, however, that disclosure of the first result and the waiting period for its confirmation are experienced in a more or less anxiety-provoking way, depending on the resources available to the parents, their ability to cope with uncertainty, their expectations, and their perception of the medical profession (Soriano, 2011).

With this in mind, after the first extensions of the NBS to conditions detectable through physiological screening, such as hearing loss, which is not immediately life threatening, or to diseases which remain life-threatening despite early treatment, such as cystic fibrosis, some researchers and clinicians (Soriano, 2011; Baroni et al., 1997; Moran et al., 2007) are questioning the real benefits of presymptomatic disclosure. They emphasize that, in the absence of any request or concern expressed by parents, such disclosure could cause significant distress (Soriano, 2011; Moran et al., 2007).

Limiting the negative effects of this early disclosure and ensuring that it does not generate more distress than a disclosure based on clinical symptoms thus becomes a major psychosocial challenge for NBS (Duff and Brownlee, 2008; Baroni et al., 1997; Parsons et al., 2002).

### 3.5 The outcome of parental distress depending on the type of result: true positive, false positive, intermediate

The final result of the screening process, obtained with additional tests carried out following a suspicious initial result, gives rise to three possible situations: true positive, false-positive, and intermediate. We observe that parental suffering caused by disclosure and its consequences for the family differ depending on the results of NBS confirmation tests.

#### 3.5.1 True positive NBS results (TP)

When a positive screening result for a newborn is confirmed through additional tests, it is referred to as a ‘true positive’. The child is or will be diagnosed with the screened disease (Tluczek et al., 2022; Duff and Brownlee, 2008; Chudleigh et al., 2016; Karaceper et al., 2016). This type of result means that the child is immediately referred for care, so that treatment can begin. Genetic counseling is systematically offered to the family. This is an opportunity for the parents and the geneticist or specialist pediatrician to discuss the etiology of the detected disease, as well as its potential implications for the child (Bordet et al., 2018). According to studies (Duff and Brownlee, 2008; Carpenter et al., 2018; Tluczek et al., 2022; Boychuk et al., 2022), this support improves understanding of the diagnosis, increases parental involvement in the child’s care from the outset, and eases their apprehension about the future. It also gives parents a sense of control over the disease (Boychuk et al., 2022), which seems to facilitate their adjustment to the situation (Carpenter et al., 2018).

Regardless of the severity of the diagnosis, studies have shown a significant reduction in parental distress in the 6 months following confirmation of the results (Kariyawasam et al., 2021), although the extent of this reduction depends on the severity of the detected disease, the efficacy of existing treatments, and parents’ perceptions of the disease and the care it requires (Duff and Brownlee, 2008; Chudleigh et al., 2016). A qualitative study (Carpenter et al., 2018) highlights the difficulty some parents of children diagnosed with phenylketonuria (PKU) have in accepting the disease and adjusting to the new routine it imposes (strict diet for the child, regular blood tests), despite the availability of treatment to prevent the onset of associated symptoms. In some cases, the authors note the existence of persistent parental grief during the child’s first months of life. This is often associated with social restriction and hypervigilance.

This condition is likely to disrupt early parent–child relationships and could have long-term consequences for the child’s psycho-affective development. Certain authors (Duff and Brownlee, 2008) have identified what they call “chronic grief” in some parents of children with cystic fibrosis, characterized by alternating periods of normality and moments of sadness, melancholy and grief, making day-to-day management more complex. A psychological study (Li et al., 2023) shows that parents’ despair, although it may have diminished at the start of care, can be reactivated during the child’s first 5 years of life, when particular medical episodes (child hospitalization) or symbolic events (child’s birthdays) occur; and cause what the authors identify as “chronic grief” requiring long-term psychosocial care for the family. The authors (Tluczek et al., 2014) of a longitudinal quantitative study note that this reactivation of parental suffering is not limited to the child’s early years.

They noted the resurgence of depressive affect in parents of children suffering from cystic fibrosis, a disease which remains life-threatening in adolescence despite early treatment. However, they found no difference in the psychosocial functioning of these adolescents as compared with their peers.

#### 3.5.2 Indeterminate, ambiguous, and inconclusive NBS results

When the additional tests carried out after an abnormal NBS result do not allow for the diagnosis to be determined with certainty, they are referred to as indeterminate, ambiguous or inconclusive results (Hayeems et al., 2017; Perobelli et al., 2009; Course and Hanks, 2019). They may be linked to the existence of a variant of the disease gene whose clinical consequences are variable or unknown (Perobelli et al., 2009), and/or to a level of biomarkers outside the normal range, but below the thresholds associated with the disease (Course and Hanks, 2019). The child is considered neither sick nor healthy after confirmatory testing, and thus falls into an intermediate zone (4). Professionals cannot predict with certainty how the child’s situation will evolve (Perobelli et al., 2009; Course and Hanks, 2019; Krishnananthan and Pao, 2019). This complicates the transmission of results to parents (Course and Hanks, 2019).

In some cases, the child will never develop the disease, but in others, mild or severe symptoms of the disease will eventually appear after a delay which varies and can be up to several years (Hayeems et al., 2017; Course and Hanks, 2019; Krishnananthan and Pao, 2019). This type of finding, which gives rise to diagnostic uncertainty for an indeterminate period, and the preventive monitoring it entails, can disrupt the family’s daily routine (Course and Hanks, 2019; Krishnananthan and Pao, 2019; Grob, 2019; Tluczek et al., 2022) and give rise to parental attitudes of hypervigilance, which can persist over time (Hayeems et al., 2017). Until a more precise diagnosis has been made, parents may perceive their child as medically vulnerable, and remain particularly attentive to the onset of any symptoms of the disease. As an example, some parents whose child has received an inconclusive result for cystic fibrosis say they are worried every time the child catches a cold or has a cough. Others actively seek out potential symptoms of the disease, regularly licking their babies to detect any increase in the salt content of their sweat, a characteristic symptom of cystic fibrosis (Hayeems et al., 2017). Parents with results of this kind are generally quite involved in their child’s care and in research protocols available to them, as this gives them access to medical experts. The relationship parents forge with professionals often seems indispensable and highly supportive (Hayeems et al., 2017). Parents’ concerns about their child’s health diminish as the child grows and they see that he or she continues to be in good health. Reassured, some then reduce medical monitoring when the child starts school, to avoid disrupting their education and being labeled as sick (Hayeems et al., 2017).

Compared with false-positive results, the psychosocial consequences of indeterminate or inconclusive results remain underexplored in the literature. The relative scarcity of empirical studies in this area should be highlighted as a significant gap. This lack of research is particularly concerning given that the integration of genomic sequencing into NBS and the expansion of screened conditions are likely to increase the frequency of such ambiguous findings.

#### 3.5.3 False-positive (FP) NBS results

As with parents confronted with indeterminate or inconclusive results, those receiving false-positive (FP) outcomes also described experiencing anxiety, uncertainty, and emotional distress. When additional tests invalidate an initially abnormal NBS result, producing a result within the expected norm for the targeted disease, this is termed a “false-positive.” In this case, the child is healthy and requires no further medical attention (Ministry of Health and Access to Care, 2024). False-positive results can be distressing for parents, as they initially suggest that their child might be seriously ill, leading to heightened anxiety and uncertainty during the period between the first and second test results (Malvagia et al., 2020; Health Resources and Services Administration, 2024).

Furthermore, additional tests performed following an initial abnormal result may reveal so-called “incidental” data, i.e., information not actively sought during screening (Lantos, 2019; Noke et al., 2014). These are not specific to FP results, and can also occur in cases of TP or indeterminate, ambiguous or inconclusive results.

In certain FP situations, such as cystic fibrosis, further testing may reveal that the child is a heterozygous carrier of a single pathogenic variant in the CFTR gene. Most carriers are asymptomatic, but in rare cases, a single pathogenic variant may cause mild symptoms, such as pancreatitis or male infertility, depending on the specific variant and individual factors. Although this genetic status does not lead to disease (to be ill, the child must carry two pathogenic variants), it may have implications for plans for pregnancy for both the parents and the child (Lantos, 2019; Noke et al., 2014; Farrell and Christopher, 2008; Vailly, 2011; Green et al., 2004). This situation raises ethical questions (Labrador Cañadas et al., 2021; Levy, 2021), as the results reveal a genetic characteristic that may lead to prenatal testing rather than a diagnosis of a disease requiring care (Vailly, 2011). As a result, routine testing of children for carrier is generally discouraged in many countries (UK, USA, Germany, etc.), although the communication of incidentally discovered carrier results remains controversial (Lantos, 2019), and how to inform parents remains a complex ethical and practical issue (Labrador Cañadas et al., 2021; Levy, 2021).

In FP situations, the parental distress caused by the initial outcome may persist despite confirmation of FP status (Quigley et al., 2018; Grob et al., 2018; Hewlett and Waisbren, 2006; Green et al., 2004). This phenomenon is often associated with the “vulnerable child syndrome” (VCS) (Grob, 2019; Labrador Cañadas et al., 2021; Farrell et al., 2011; Farrell et al., 2020a,b), a concept introduced by Green and Solnit (1964). This syndrome is not specific to NBS or FP results; it can also occur in cases of TP and indeterminate, ambiguous or inconclusive results, although it has not been identified as a central psychosocial issue in these situations by the dedicated literature. VCS refers to the impact of the discovery of a serious, lifethreatening illness on parents’ perception of their child’s vulnerability. When parents anticipate a lifethreatening outcome, some of them develop this syndrome, which manifests itself in excessive and persistent worry about the child’s health, even after recovery (Green and Solnit, 1964), including the fear that the child will develop the disease later (Quigley et al., 2018; Karaceper et al., 2016; Duff and Brownlee, 2008; Tluczek et al., 2011; Farrell et al., 2020a,b; Grosse et al., 2009). In the case of false-positive NBS results where children are diagnosed only as heterozygotes (e.g., cystic fibrosis or sickle cell disease screening), one study revealed that 7.8% of parents remained convinced that their child was still at risk of developing the disease in the future (Farrell et al., 2020a,b). These findings are corroborated by a longitudinal study which indicates that 23% of parents continue to fear that there was a misdiagnosis at birth every time their child develops respiratory symptoms (Farrell et al., 2020a,b).

VCS can have significant repercussions on the child and his or her family. Parental skepticism about the results (Quigley et al., 2018; Farrell et al., 2020a,b) can lead to hypervigilance (Duff and Brownlee, 2008; Farrell et al., 2020a,b; Perobelli et al., 2009) and overprotection, sometimes resulting in overmedicalization of the child (Karaceper et al., 2016; Farrell et al., 2020a,b; Grob et al., 2018; Grosse et al., 2009; Hewlett and Waisbren, 2006). One study found that children with a FP result were three times more likely to be hospitalized than those with a normal NBS result (Tu et al., 2012), while another study (Lipstein et al., 2009) found no difference between these two groups.

Some researchers (Karaceper et al., 2016) suggest that these parental behaviors of hypervigilance and overprotection, characteristic of VCS, may have more to do with the prematurity of the children, often over-represented in FP results, than with an initial abnormal NBS result.

When these behaviors persist, they are likely to affect the parent–child relationship (Tluczek et al., 2022; Hewlett and Waisbren, 2006) and the attachment process (Duff and Brownlee, 2008), with potential negative consequences for the child’s autonomy (Carpenter et al., 2018; Quigley et al., 2018; Tluczek et al., 2022) and psycho-affective development (Carpenter et al., 2018; Campbell and Ross, 2003; La Pean and Farrell, 2005; Farrell et al., 2005; Farrell et al., 2011). Perceived as ill by those around them (Farrell et al., 2020a,b), the child may also be stigmatized or discriminated against (Duff and Brownlee, 2008; Noke et al., 2014).

Thus, the NBS program may cause significant medical and psychological harm to a small number of screened children and their families (Rothenberg and Sills, 1968; Goldenberg et al., 2016; Hewlett and Waisbren, 2006), without offering any medical benefit. FP results can also pose organizational challenges, as VCS leads to over-consumption of health services, with the risk of saturating the care system when the child is not ill (Karaceper et al., 2016; Farrell et al., 2020a,b; Grob et al., 2018; Hewlett and Waisbren, 2006).

### 3.6 Risk factors for negative psychosocial impacts, including vulnerable child syndrome (VCS): parents’ difficulty in understanding NBS results delivered by healthcare professionals

Parents’ lack of understanding of NBS results appears to be a determining factor in the risk of VCS. This is due to ineffective communication between doctors and parents, often as a result of: insufficient training of healthcare professionals in the transmission to parents of NBS results, particularly FPs; parents’ low level of genetic knowledge, which limits their understanding of NBS results; and, finally, the poor quality of interactions between doctors and parents at key moments in the screening process.

#### 3.6.1 Ineffective transmission of information by doctors (FP cases used here as illustrative examples)

Numerous studies (Tluczek et al., 2022; Carpenter et al., 2018; Hewlett and Waisbren, 2006; Farrell and Christopher, 2008; Farrell et al., 2005; La Pean and Farrell, 2005; Farrell et al., 2011) have found that the way in which doctors convey information about a NBS FP result can be confusing for parents and affect their understanding of the results.

A medical team (Farrell and Christopher, 2008; Farrell et al., 2005; La Pean and Farrell, 2005) looked at the vocabulary used by doctors during disclosure consultations, the types of information delivered, and the points in the interview at which this information was transmitted. To this end, they devised a research protocol based on scenarios in which young doctors were asked to announce a FP result for cystic fibrosis or sickle cell disease to fictitious standardized parents (Farrell and Christopher, 2008; Farrell et al., 2005; La Pean and Farrell, 2005). The results showed the use of medical vocabulary that was poorly adapted to their audience and not explained during the consultation (Farrell and Christopher, 2008).

What’s more, the information conveyed was often ambiguous, as it concerned disease progression and outcomes and led parents to understand that their child was ill when he was not (Farrell et al., 2005). Although in the majority of cases, the child’s non-disease status was confirmed during the interview, the authors warn of the confusion that can be generated by an excess of useless information. They also point to the primacy effect of the disclosure, meaning that parents tend to remember only the first message delivered to them. In reality, participants can be exposed to considerable anxiety, as well as a high risk of developing serious psychosocial complications, due to poor communication by professionals (Farrell et al., 2005; La Pean and Farrell, 2005).

In this context, FP cases are presented solely to illustrate how ineffective communication by professionals can generate considerable anxiety and increase the risk of psychosocial complications, rather than as the main focus of this review.

#### 3.6.2 A lack of knowledge about NBS in the general population

According to several studies (Baroni et al., 1997; Blom et al., 2021; Green et al., 2004), parents’ difficulty in understanding NBS results may be exacerbated by a general lack of medical knowledge, particularly about NBS, as well as about how genetic diseases are transmitted (Tluczek et al., 2022). It seems, however, that this theoretical knowledge deficit can be overcome by experiential knowledge of the disease. Studies (Noke et al., 2014; Tluczek et al., 2005) have found that parents with prior knowledge of the disease being screened, either because of its frequency in the general population, or because of its presence in their family, friends, or professional environment, are better able to understand NBS results (Noke et al., 2014). This familiarity with the disease plays a protective role for the parental couple when an abnormal NBS result is announced (Hayeems et al., 2016; Tluczek et al., 2005). The ability to anticipate future events thanks to this lived experience reduces the level of distress during disclosure and confirmation of the result (Chudleigh et al., 2016; Boychuk et al., 2022) and favors the management of emotions and the parents’ adjustment to the child’s illness (Tluczek et al., 2022; Duff and Brownlee, 2008).

#### 3.6.3 Negative perception of the healthcare professional-parent interaction

Another dimension that seems to play a decisive role in parents’ understanding of the results is the way they perceive their interactions with professionals at three key moments in the screening process: the sampling, the initial disclosure of NBS results, and the disclosure of the confirmation of the results (Chudleigh et al., 2016).

The distress caused to parents by a positive result is greater if they feel they were not clearly informed at the time of sampling that the child would be screened, or of the possible implications (Chudleigh et al., 2016).

Studies (Tluczek et al., 2022; Chudleigh et al., 2016; Farrell et al., 2020a,b) have shown that parental anxiety is heightened when the initial disclosure is made by a healthcare professional not specializing in NBS (private nurse, general practitioner), who is often unable to answer their questions (Tluczek et al., 2022; Chudleigh et al., 2016; Farrell et al., 2020a,b). In FP situations, when the re-test invalidates the initial result, the professional’s difficulty in explaining the final result can increase the parents’ anxiety (Tluczek et al., 2022; Chudleigh et al., 2016; Farrell and Christopher, 2008).

This heightened anxiety and confusion is a key component of what has emerged in our analysis as the central psychosocial risk: VCS (Vulnerable Child Syndrome). Thus, while parental misunderstanding contributes, it is the interaction between the parents’ distress and the professional’s communication that often triggers VCS, which then becomes a primary lens through which we observe negative psychosocial outcomes.

Negatively perceived interactions with professionals may have long-term consequences for the healthcare relationship. They are likely to generate distrust of medical institutions (Chudleigh et al., 2016), promote a negative perception of the NBS procedure, weaken parents’ commitment to care and limit their participation in screening in the future (Soriano, 2011; Duff and Brownlee, 2008).

The quality of this interaction could also have an impact on the child’s subsequent medical care. According to this study (Karaceper et al., 2016), in the first few years of a child’s life, doctors tend to hospitalize infants with FP NBS results more frequently, especially when a rare and serious disease was initially suspected, such as Medium-chain Acyl-COA Dehydrogenase Deficiency (MCADD), a metabolic pathology that can lead to hypoglycemia and even heart rhythm disorders. This tendency is further reinforced when parents are anxious and confused about the final results of the NBS. According to this study, the doctor’s perception of the illness (which the child does not have) and the parents’ distress may play a decisive role in the decision to hospitalize the child. Thus, in this context, the overmedicalization characteristic of VCS may be more the result of the interaction between the doctor and the parents, rather than simply of the parents’ misunderstanding of the results, highlighting why VCS emerges as a central organizing concept in understanding negative psychosocial impacts.

### 3.7 Axis 2 avenues and actions to limit the psychosocial risks associated with NBS: education for professionals and the general population

Training healthcare professionals (Farrell and Christopher, 2008; Farrell et al., 2005; La Pean and Farrell, 2005; Hewlett and Waisbren, 2006) to improve communication when results are disclosed, and providing the general population with information on screening (through media such as videos, websites, brochures and meetings with professionals), appeared to be essential in reducing the psychosocial risks associated with these results at a low cost (Pàmpols Ros et al., 2022; Bailey et al., 2009; Quigley et al., 2018; Farrell and Kuruvilla, 2008; Tu et al., 2012; Prakash et al., 2022; Araia et al., 2012; Hewlett and Waisbren, 2006).

### 3.8 Education for professionals and the general public

#### 3.8.1 Professional training

Research (Farrell and Christopher, 2008; Farrell et al., 2005; La Pean and Farrell, 2005; Farrell and Kuruvilla, 2008) into the quality of communication between healthcare professionals and parents recommends training practitioners who need it most, in order to improve the quality of disclosure consultations and to ensure better understanding of results by parents. For FP results, authors recommend following a standardized procedure: because of the primacy effect identified by researchers, the consultation should systematically begin with the good news (“the child is not ill”) (La Pean and Farrell, 2005). In the case of inconclusive results, which present different uncertainties for parents, one study (Perobelli et al., 2009) similarly suggests that disclosure should start with a clear message that the infant’s situation is currently benign, while keeping detailed information about potential etiology and consequences to a minimum (Perobelli et al., 2009).

The authors (Farrell and Kuruvilla, 2008) also suggest standard phrases to facilitate exchanges between professionals and parents. The aim of such training is to guarantee “ideal” clinical behavior and clear transmission of information, while ensuring that parents fully understand. This would help to resolve the ethical problems raised by FP and inconclusive situations, often cited as an argument against the extension of neonatal screening (La Pean and Farrell, 2005).

This raises the question however, as to how this complex and constantly evolving training should be integrated, given the ever-growing list of diseases to be screened. It is also important to determine what form training should take (Grob, 2019).

#### 3.8.2 Information for the general public

Researchers also recommend providing information to the general population about NBS, to improve parents’ understanding of the results at the disclosure consultation. They suggest access to information online via a website (Pàmpols Ros et al., 2022; Duff and Brownlee, 2008). However, studies (Tluczek et al., 2022; Bailey et al., 2009) show that parents’ knowledge improves when information is communicated by a competent professional orally, followed by a written document, while online resources can be overwhelming as parents are not always able to parse and prioritize the information. Access to written information online is therefore no substitute for a well-informed medical expert (Bailey et al., 2009). This is particularly true for people with a level of reading comprehension below the population average.

In the latter case, oral communication is often more effective. Viewing a video could be an appropriate alternative to the presence of a professional (Quigley et al., 2018). However, the level of knowledge of genetics is particularly low in the general population and varies between individuals. This raises the question as to how to adapt content to socio-economic, linguistic and educational inequalities (Tluczek et al., 2022; Pàmpols Ros et al., 2022), so that it is both comprehensive and accessible to all (Bailey et al., 2009). Some authors (Green et al., 2004) point out that informative materials, while slightly improving knowledge, do not guarantee sufficient understanding to make informed decisions about screening, nor do they reduce the risks associated with a FP NBS result. They warn against the danger of substituting informational aids for consultation with a professional in order to cut costs, in the context of rapid and unprecedented expansion of NBS.

Some researchers (Grosse et al., 2004; Baroni et al., 1997; O'Connor et al., 2018) suggest that families who receive an abnormal NBS result should systematically have access to GC. One study (Chudleigh et al., 2016) shows that when parents faced with a FP NBS result receive GC or are able to consult professionals specialized in this screening, their understanding of the results improves. They are less anxious, the dialogue is more fluid, and they are better able to develop a strong bond with the child following the disclosure. The risk of developing VCS is thus greatly reduced (Chudleigh et al., 2016; Parsons and Bradley, 2003; Baroni et al., 1997).

However, these results have been qualified by another study identified in a literature review, which showed that some mothers of children who are heterozygous carriers of cystic fibrosis or sickle-cell anemia retain only a limited understanding of the result after GC due to persistent anxiety (Tluczek et al., 2022).

#### 3.8.3 The limits of training in reducing the psychosocial risks associated with NBS in the context of its unprecedented expansion

Given the complexity of the information provided by extended NBS, training for both professionals and the general population may not be sufficient to ensure adequate understanding of the results at the initial consultation, nor to ensure truly informed consent for the screening itself. Integrating NBS into the care process early by professionals known to patients in private practice, as is advocated by certain professionals and associations, now seems essential (Labrador Cañadas et al., 2021; Tluczek et al., 2022; Chien and Hwu, 2023; Blom et al., 2021). In addition to integrating the practice of screening and the risk of a positive result into the care relationship, it would also allow parents to be truly involved in the decision of whether or not to participate (Pàmpols Ros et al., 2022).

This could be one way of ensuring that parents participating in neonatal screening (Labrador Cañadas et al., 2021) are giving truly informed consent. It could also help reduce the distress caused by an initial positive result by preparing parents for this possibility. Conceptualizing the practice of screening over a relatively long timeframe, rather than reducing it to the disclosure of results, seems essential in light of the inevitable increase in the complexity of information to be provided to parents (Labrador Cañadas et al., 2021; Bensimon and Araneda, 2022).

### 3.9 Information provided within the context of a dynamic, interactive relationship

Although few studies have specifically examined how information is delivered within a dynamic, interactive relationship between healthcare professionals and parents, these works provide particularly rich and nuanced insights and are therefore discussed in detail.

In order to ensure effective transmission of information, the healthcare professional must adapt to each specific set of parents during disclosure consultations. Faced with the significant anxiety provoked by the initial disclosure of an abnormal screening, parents adopt a variety of complex coping strategies to which the professional must pay close attention (Dillard and Carson, 2005). Some parents seek information about the disease, and want to know about all the procedures associated with the screening in detail, in order to reduce the uncertainty, they find distressing. In contrast, others prefer to avoid information as an effort to preserve uncertainty, which they perceive as protective in the face of what they fear to be a particularly unfavorable outcome. These parents may prolong this uncertainty well beyond the confirmation of the diagnosis. If a doctor provides information about genetic risks without taking into account the parents’ particular strategy around uncertainty, it can lead to major psychosocial complications for the child and his or her family.

The authors of the study (Dillard and Carson, 2005) therefore insist that parents and professionals must be able to negotiate the limits of the discussion during these disclosure consultations. It is also crucial for professionals to remember that anxiety is an appropriate parental response to a threat to their child’s health, and that attempting to eliminate it completely would be unrealistic (Green et al., 2004). In addition, a certain level of anxiety can be beneficial in helping parents to focus on the essential parts of the information they are given, sometimes improving their understanding of the results and positively influencing their decisionmaking regarding future treatments (Green et al., 2004).

An overly rigid standardization of disclosure which forces practitioners to systematically deliver certain information and key messages, could frustrate parents and interfere with the various coping strategies they adopt. It could also undermine the collaborative effects at work between patients and doctors (Dillard and Carson, 2005). It is therefore important to provide professionals with guidelines to help them communicate NBS results to families, while ensuring that the protocol remains sufficiently flexible to allow them to adapt to the complex strategies of their audience (Dillard and Carson, 2005).

The quality of this adaptation is a determining factor not only in families’ understanding of NBS results, but also in their experience of the screening overall. Successful adaptation strengthens parents’ confidence in the healthcare professional, facilitates their acceptance of the diagnosis, and makes it easier to dispel any doubts about the child’s health in the event of a FP result. The trust built can also improve the perception of the healthcare system and encourage parents to comply with NBS (Chudleigh et al., 2016; Soriano, 2011; Duff and Brownlee, 2008; Moran et al., 2007).

Authors (Blom et al., 2021; Pàmpols Ros et al., 2022) note that this adaptation is facilitated when parents interact with professionals over the course of a long-term caring relationship. Conversely, a disclosure made by a professional unknown to the family increases parents’ anxiety, and additional interlocutors during this period alters their confidence in the outcome and in the care system (Soriano, 2011; Chudleigh et al., 2016). This is why many professionals, as well as parents’ and patients’ associations, advocate that information about screening and its potential consequences should be given earlier in pregnancy (Labrador Cañadas et al., 2021; Tluczek et al., 2022; Chien and Hwu, 2023; Blom et al., 2021; Duff and Brownlee, 2008), by professionals in private practice (general practitioners, gynecologists, midwives) already known to the families (Tluczek et al., 2022; Blom et al., 2021; Duff and Brownlee, 2008) and with whom they have a long-term care relationship.

### 3.10 Axis 3 the challenges of extending NBS

Thanks to recent scientific advances in genomic medicine, the integration of genome sequencing into NBS is becoming a reality (Chien and Hwu, 2023; Tluczek et al., 2022; Grob, 2019). This may change the kind of diseases which are screened for, thus redefining the objectives of screening. This evolution may change not only the profile of individuals screened, but also the healthcare relationship. The expansion of NBS therefore raises new ethical and psychosocial issues, making informed public acceptance all the more crucial.

### 3.11 «Patients in waiting»: a new category of patients driving changes in the care relationship

The integration of genome sequencing with NBS could make it possible to identify all of an individual’s genetic variants from birth, considerably increasing the initial number of abnormal results (Pàmpols Ros et al., 2022). While this technique promises to reduce false-positive results for well-known diseases, it is likely to generate numerous FP or indeterminate results for rarer and less understood pathologies, as well as for heterozygous carriers and carriers of variants of low severity or unknown significance (Vailly, 2011; Levy, 2021). Experience also shows that each extension of NBS to new diseases is systematically accompanied by incidental data (Course and Hanks, 2019). For example, the recent inclusion of Pompe disease in certain screening programs has made it possible to detect not only its early-onset form, but also the not-sought out, late-onset version (LOPD) (Prakash et al., 2022; Crossen et al., 2022; Pruniski et al., 2018).

In the same vein, the extension of NBS may frequently identify genetic alterations associated with late onset diseases, or genetic diseases for which no treatment exists (Labrador Cañadas et al., 2021; Levy, 2021). The extension of NBS would then be deviating from its original purpose (Labrador Cañadas et al., 2021; Levy, 2021) of saving lives and improving life expectancy for children (Grob, 2019; Bailey et al., 2009), by gradually abandoning its origins in urgency (Bailey et al., 2009) and seeking to reduce diagnostic delay without bringing any direct benefit to the child (diagnosis of an untreatable disease or a disease whose first symptoms occur in adulthood).

This development could accelerate the emergence of a new category of patient, referred to as “patients in-waiting” (Grob, 2019; Hayeems et al., 2017; Boychuk et al., 2022; Crossen et al., 2022; Lisi and Ali, 2021; Pruniski et al., 2018), a concept introduced by Timmermans and Buchbinder (2010) to designate asymptomatic individuals labeled as “patients” solely because of screening practices. These people, who find themselves between health and disease live in uncertainty, waiting for an illness that may never materialize (Crossen et al., 2022; Lisi and Ali, 2021; Pruniski et al., 2018). In the case of LOPD, for example, the person knows that he or she will be affected by the late-onset form of the disease but does not know when symptoms will appear (usually between the ages of 10 and 60) (Prakash et al., 2022; Dillard and Carson, 2005; Crossen et al., 2022).

Asymptomatic individuals, thus centered in the healthcare system by the extension of NBS, could see their lives profoundly impacted. Uncertain results and the identification of late-onset diseases demand prolonged medical observation in order to establish a diagnosis, regularly screen for the appearance of symptoms, and/or prevent possible complications (Timmermans and Buchbinder, 2010).

The majority of parents questioned in these studies support including these pathologies in NBS and highlight the non-healthcare benefits doing so may bring them (Chung et al., 2016; Goldenberg et al., 2016; Qian et al., 2015). They feel that it could prevent the obstacles to diagnosis, and reduce the uncertainty, worry, and guilt, as well as the financial cost, associated with these diseases (Tluczek et al., 2022; Goldenberg et al., 2016; Qian et al., 2015; Hayes et al., 2007). For them, knowing about the disease before the first symptoms appear would mean being better prepared for the future: prioritizing quality time with the child, planning for career or lifestyle changes before the first signs of disease so as to be more available to their child, identifying specialized schools where the child could continue their education when symptoms become disabling, educating family and friends about the disease early on (Campbell and Ross, 2003), and making informed decisions about future pregnancies (Tluczek et al., 2022; Chung et al., 2016; Parsons et al., 2002; Hayes et al., 2007). This personal utility outweighs clinical utility for them (Levy, 2021), explaining why parents are more supportive than professionals of screening for incurable and late-onset diseases (Labrador Cañadas et al., 2021; Campbell and Ross, 2003).

Several authors (Grob, 2019; Bush et al., 2022; Crossen et al., 2022; Lisi and Ali, 2021; Pruniski et al., 2018) warn however, that the emergence of this new category of patients could lead to a resurgence of certain problems that NBS was designed to limit, such as diagnostic uncertainty. Indeed, expanded NBS could well produce new forms of uncertainty and lead to a resurgence of the diagnostic odyssey (Grob, 2019; Bush et al., 2022). As we have already mentioned, waiting for the confirmation of results can increase parental anxiety and stress, and give rise to attitudes of hypervigilance that can ultimately complicate the child’s psycho-affective development (Prakash et al., 2022; Crossen et al., 2022; Pruniski et al., 2018; Lisi and Ali, 2021).

### 3.12 Consequences of this new category of “patients in waiting” for healthcare systems

The emergence and categorization of “patients in waiting” (Timmermans and Buchbinder, 2010) as a new patient group will inevitably have repercussions for healthcare systems. While the extension of NBS has its advantages, it also poses significant challenges (Pàmpols Ros et al., 2022; Levy, 2021; Kölbel et al., 2022). Indeterminate results and the identification of late-onset diseases necessitate prolonged medical observation (Timmermans and Buchbinder, 2010), which can lead to over-treatment, particularly when benign forms of little-known diseases are detected. Similar situations have been observed in the past with diseases such as severe combined immunodeficiency (SCID), or phenylketonuria (PKU) (Chien and Hwu, 2023). On the other hand, extended NBS could not only benefit the screened child and their family, but also prevent the onset of diseases in future generations by encouraging the use of prenatal and preimplantation screening (PND, PGD) (Vailly, 2011).

Such developments in NBS raise important ethical questions, particularly with regard to consent. In a context of unprecedented scientific advances, it is becoming difficult for parents to fully understand what they are consenting to at the time of collection. It is impossible to anticipate what future discoveries might be made from these samples (Lantos, 2019). This situation is particularly worrying in countries such as the USA, where private laboratories are authorized to carry out these tests and analyze samples, opening the door to potential misconduct (Bailey et al., 2009; Le Driant et al., 2006; Hewlett and Waisbren, 2006). Some parents fear that the samples will be used without their consent (Hewlett and Waisbren, 2006), or that their adult child may be denied access to certain important services (such as insurance or loans) on the basis of NBS results (Lisi and Ali, 2021; Hewlett and Waisbren, 2006).

Lastly, some researchers (Goldenberg et al., 2016; Grob, 2019) warn that these indeterminate results and the identification of late-onset diseases may exacerbate inequalities. Although all children would be screened, access to appropriate care would likely depend on where they live and their socio-economic background. The regular monitoring required by these results can represent a substantial cost for families.

Some authors (Grob, 2019; Hewlett and Waisbren, 2006) fear that the extension of NBS, combined with a lack of appropriate care for families, could undermine the entire screening program. The emergence of this new category of patients is therefore bound to have repercussions on healthcare systems.

### 3.13 A new paradigm in healthcare

Genomic NBS could put an end to the classic dichotomy between health and illness, in favor of a continuum on which the screened child would be positioned (Labrador Cañadas et al., 2021; Levy, 2021).

The inclusion of genome sequencing in NBS could lead to a two-tiered screening system: the first limited to disease genes which are treatable, or for which action can be taken, and the second, providing potentially useful medical information for the future (Lantos, 2019). This could redefine the very notion of health, by encompassing individual genetic knowledge. We are therefore at the dawn of a new paradigm based on prediction, where the potential psychosocial risks associated with NBS could be offset by the prospect of future medical advances (Lantos, 2019). In this context, informed public acceptance of NBS becomes a major psychosocial issue, essential to its successful extension.

## 4 Discussion

Building on the descriptive synthesis presented in the Results section, the following discussion moves toward a higher level of conceptualization. While the Results section closely reflected the empirical findings derived from the reviewed studies, the Discussion develops a more interpretive analysis that situates these findings within broader psychosocial and theoretical frameworks. Table illustrates this analytical progression, showing how the thematic axes identified through inductive analysis informed the higher-order conceptual categories developed in the Discussion. The three descriptive axes from the Results section were further interpreted and elaborated into three psychosocial figures, reflecting a higher level of conceptualization (Table 7).

**Table 7:** Empirical axes from results and corresponding conceptual themes in the discussion.

Empirical axes and themes identified from data (Results section)	Corresponding conceptual axes developed in the Discussion
Axis 1. Parents’ experience of receiving an abnormal screening result and its impact on their relationship with the child (e.g., distress, misunderstanding, false positives)	Psychosocial Figure 1. Anxious and depressive dimensions of parents’ emotional responses
Axis 2. Avenues and actions to limit psychosocial risks: education and information for professionals and the general public	Psychosocial Figure 2. Vulnerable Child Syndrome as a psychosocial construct shaping parental perceptions and care practices
Axis 3. Challenges of expanding NBS and emergence of “patients in waiting”	Psychosocial Figure 3. Challenges and psychosocial implications of expanding neonatal screening

This analytical mapping illustrates how the descriptive findings presented in the Results section were interpreted and synthesized into broader conceptual frameworks. Each conceptual axis thus extends beyond the empirical data to highlight the psychological, relational, and ethical dynamics underlying the psychosocial impact of neonatal screening.

Building on these conceptual axes, this literature review has highlighted the many psychosocial facets associated with newborn screening (NBS). Over time, psychosocial considerations have emerged as an essential criterion, alongside medical and financial considerations, for the evaluation of any expansion of NBS. The emergence of this in the literature, from the late 1990s onwards, has coincided with the first loosening of the Wilson and Jungner criteria (Grob, 2019; Pàmpols Ros et al., 2022; Labrador Cañadas et al., 2021), aimed in particular at maintaining strong public support for NBS, which had previously been indisputable (La Pean and Farrell, 2005; Green et al., 2004).

### 4.1 Axe 1: anxious and depressive aspects of the parents’ emotional state

Our results reveal that anxious and depressive symptoms are the most frequently studied psychosocial variables, with particular attention paid to the moment of disclosure of NBS results. This moment is often perceived as one of biographical disruption in parents’ lives. Researchers aim to ensure that the disclosure of NBS results does not generate more anxiety, depression, or worry than a post symptomatic disclosure (Soriano, 2011). However, it is important to note that assessment of these disorders is generally carried out in the short to medium term, limiting the understanding of the long-term impact of the disclosure on the parent–child relationship (Tluczek et al., 2022).

Moreover, the complex emotions of parents confronted with this experience may not be fully captured by standard measures of anxiety and depression used in mostly quantitative studies (Tluczek et al., 2022; Parsons et al., 2003; Grob, 2019), potentially leading to an underestimation of parental suffering (Grob, 2019; Tluczek et al., 2022; Parsons et al., 2003; Green et al., 2004). As analysis methods of these tools tends to focus on averages reflecting the experience of the majority of participants, they inherently do not account for extreme experiences lived by a small number of participants (Grob, 2019; Green et al., 2004). It is also likely that these studies primarily included the most cooperative parents: those who had a positive experience of screening because their child benefited from it for his or her health, or those whose child was not screened but could have benefited from it. On the other hand, those who would be most negatively affected by NBS are those who are likely to choose not to participate (Tluczek et al., 2022; Grob, 2019).

Finally, studies on the disclosure of positive and indeterminate results remain rare, as do those exploring the attachment process between parent and child and the influence of NBS results (positive, false-positive, indeterminate) on the couple’s relationship, particularly when the detected disease has been transmitted by one or both parents. Another variable worth exploring is parental attitudes to subsequent pregnancy. It is crucial to understand whether and how the results of the NBS influence their reproductive decisions. This dimension, largely absent from the current literature, could reveal profound psychosocial implications, influencing not only family dynamics, but also the reproductive choices of parents faced with uncertain or alarming NBS results.

The study of the short- and medium-term psychosocial impact on parents therefore seems essential in the current framework of NBS and its extension. In the absence of curative treatments or immediate preventative measures for some of the diseases detected, NBS results could generate significant new issues within families. A thorough understanding of these issues is essential to developing appropriate support strategies (Kölbel et al., 2022). Research in the humanities and social sciences (HSS) plays a crucial role here, providing the necessary foundations for the implementation of targeted psychosocial support modalities, responding to the specific needs of parents faced with these complex situations.

With the implementation of this type of study, we will not only be able to improve support for families, but also encourage a more holistic and ethical approach to NBS, which takes into account human dimensions as much as medical considerations.

### 4.2 Axe 2: vulnerable child syndrome

Our results indicate that VCS is the main psychosocial risk associated with NBS, particularly in the case of FP results. In this case, VCS is not counterbalanced by a medical benefit. This excessive parental concern for the child’s health may not only complicate his or her psychosocial development, but also saturate health care systems and therefore, be an argument for limiting the extension of NBS to new diseases (Farrell and Christopher, 2008; Noke et al., 2014; Farrell et al., 2011; Van Dijk et al., 2021; Blom et al., 2021). Researchers attribute this phenomenon to parents’ poor understanding of the results. They therefore stress the importance of educating professionals and the general public about screening in order to improve doctor-parent communication at the time of disclosure and reduce the risk of VCS (Van Dijk et al., 2021; Noke et al., 2014; Blom et al., 2021; Farrell and Christopher, 2008; Farrell et al., 2011).

None of the studies included in our scoping review, however, address the implementation of these measures by the various screening programs or assess their effectiveness, raising the legitimate concern of their relevance in limiting the risk of VCS with an unprecedented extension of NBS. The integration of genomic sequencing into NBS is likely to change the kind of diseases which are screened, leading to an increase in the number of abnormal results and making the transmission of results more complex, with the risk of further weakening interactions between professionals and patients. The results of our review coincide with two recent studies, focused on the impact of uncertain results on the parents of the tested child. These researchers show that parents often interpret these results with a traditional medical model (centered on the identification of symptoms and the formulation of a diagnosis), which does not allow them to navigate through this ambiguous situation. The authors point out that this is an ontological change in models and they propose to reflect on the bridging work necessary for professionals to support parents in the transition toward a model adapted to the assimilation of information about uncertainty (Johnson et al., 2019; Johnson et al., 2022).^1^

Finally, we may wonder whether these measures will be sufficient to limit the risk of VCS in a context of NBS expansion. Indeed, studies in the medical sciences are over-represented compared with those in the social sciences, and the psychological impact of such disclosure is often overlooked. It seems important that psychology researchers take an interest in the psychosocial implications of NBS in the future.

### 4.3 Axe 3: challenges and psychosocial implications of expanding neonatal screening

With the extension of screening, a significant number of new patients will experience a positive test result in the years to come (Grob et al., 2018). The potential parental distress associated with screening for certain diseases (incurable, untreatable) will be less and less justified by the corresponding medical benefit.

In this context, public acceptance of NBS becomes a major challenge for its extension. The majority of studies in hypothetical situations conclude that there is strong public support for its extension, suggesting that all parents wish to have information about screening, and that they have a sufficient degree of tolerance of uncertainty to allow them to cope with potentially uncertain results (Grob, 2019). Authors (Green et al., 2004; Campbell and Ross, 2003; Labrador Cañadas et al., 2021; Tluczek et al., 2022; Grob, 2019) nevertheless urge caution, as the generalization of this strong parental support for NBS may well be limited for methodological reasons. In particular, Measurement scales and questionnaires may fail to capture parents’ sometimes ambivalent stance toward NBS (Grob, 2019). Moreover, many of the studies identified in the literature are based on hypothetical scenarios, particularly in cases when the disease is not yet included in the screening program and is the subject of a pilot study. Participants are asked to imagine themselves as parents of infants being offered an extended version of NBS, or as parents of a child with an abnormal screening result, even though they have never experienced such a situation. The authors note that participants in these studies are likely to be better informed about NBS than the average person who is actually offered this kind of screening. Therefore, the context in which the hypothetical decision to pursue NBS or not is made does not necessarily correspond to the real-life scenario which a parent in the general population would experience (Green et al., 2004).

In addition, based on the dedicated literature, the authors demonstrate that decision-making differs when using theoretical intellectual versus experiential knowledge. Indeed, the identification of a child with cystic fibrosis through NBS would have a limited impact on the parents’ subsequent reproductive behavior, whereas a diagnosis based on the child’s clinical symptoms would limit future pregnancies (Green et al., 2004). This idea is supported by Smith et al. (cited by Campbell and Ross, 2003), who questions the strong public support for extended NBS to incurable or late-onset diseases, placing it in the context of other forms of screening, particularly in adult clinics. Very few adults offered presymptomatic screening for diseases without effective treatments or prevention (such as Huntington’s disease, or certain forms of breast cancer) go through with it.

When no medical intervention is available, most individuals prefer not to seek this information. Smith et al. (Campbell and Ross, 2003) therefore hypothesize that parents supporting extended NBS in studies, may hold the firm belief that exploratory screening implies some potential treatment. According to the authors, participants’ response in these studies may reflect the mistaken idea that research necessarily has a therapeutic purpose, which could explain the high level of public support for extended NBS observed in the literature.

It has also been noted (Labrador Cañadas et al., 2021; Tluczek et al., 2022; Grob, 2019; Green et al., 2004) that these studies probably misrepresent parents from the general population, particularly as they are not organized into identifiable groups (Labrador Cañadas et al., 2021). The participants in these studies would essentially be parents seeking out NBS and/or having benefited from it, or else participants who have never actually been confronted by it and/or never experienced an abnormal result (Tluczek et al., 2022; Grob, 2019; Green et al., 2004).

Viewing the practice of screening as a real process, beginning well before any sample is taken and forming part of a care relationship with independent healthcare professionals, could be a promising way of limiting the risks associated with a positive screening result. The care relationship could also resolve some of the major issues relating to informed consent of parents undergoing NBS. According to Ricoeur, true consent cannot be obtained without building a long-term relationship of trust. He refers to this as the “path of consent” (Ricoeur, 1990). However, in the current NBS context, it seems that consent is reduced to a lack of opposition to sampling.

While previous experience, particularly with cystic fibrosis, which has given rise to a great deal of research, allows anticipation of some psychosocial issues that the unprecedented expansion of NBS might raise, it also introduces new challenges in terms of care and consent. This expansion could profoundly transform the healthcare relationship. Previous experiences of screening may not be sufficient to fully understand what is about to happen in NBS. New research is needed to better anticipate the risks associated with extended screening.

## 5 Limits

The results of this study must take into account certain limitations. The bibliographical search was restricted to three databases. The absence of articles dealing with this issue prior to 1997 may well be linked to the fact that psychosocial journals have not digitized or indexed articles prior to this date. In addition, ethical aspects which are strongly linked to psychosocial issues, do not appear much in our results, possibly as a result of the limited number of keywords used in our search.

## 6 Strengths of the study

This review spans a wide time period, draws on multiple disciplines (including the social sciences and humanities), and is grounded in an interdisciplinary, collaborative analytic process. Its inductive approach, not limited to a single pathology, allows the generation of hypotheses that may be applicable across different clinical contexts.

## 7 Conclusion

Upon completion of this research, we have identified three psychosocial figures which define NBS: anxious and depressive aspects of the parents’ emotional state, vulnerable child syndrome, challenges and psychosocial implications of expanding neonatal screening. These psychosocial constructs have evolved over time in interaction with the major technological and social changes that have taken place alongside NBS over the last few decades. Nevertheless, they have largely been shaped by FP outcomes in a particular pathology. As such, they may not be sufficient to address the psychosocial challenges posed by the unprecedented expansion of NBS. Indeed, it is likely that the progressive integration of genome sequencing will generate a significant amount of incidental data and ambiguous or uncertain results, which could radically alter the psychosocial dimension of screening. At this stage in our current experience of screening, it is difficult to grasp the scale of this phenomenon and therefore its potential impact on healthcare systems and the individuals taking part in it. Thinking of NBS as a process, and integrating this practice within a genuine healthcare relationship, seems nevertheless to be particularly relevant to preventing the complex psychosocial risks it is likely to cause.

These risks cannot be reduced to the issue of parents’ potential understanding of results and/or communication with healthcare professionals. However, further research in the fields of social sciences and the humanities is essential. In the course of this scoping review, we have noted an overrepresentation of quantitative studies from the medical sciences regarding the psychosocial aspects of NBS, while qualitative and clinical research in the Human and Social Sciences remains underrepresented, despite it falling explicitly within this domain.

## Data Availability

The original contributions presented in the study are included in the article/Supplementary material, further inquiries can be directed to the corresponding author.

## Footnotes

1 These references were added following the peer review process.
